# Improved Outcome of Biliary Atresia with Postoperative High-Dose Steroid

**DOI:** 10.1155/2013/902431

**Published:** 2013-11-24

**Authors:** Rui Dong, Zai Song, Gong Chen, Shan Zheng, Xian-min Xiao

**Affiliations:** Department of Pediatric Surgery, Children's Hospital of Fudan University, and Key Laboratory of Neonatal Disease, Ministry of Health, 399 Wan Yuan Road, Shanghai 201102, China

## Abstract

*Objective*. The dosage, duration, and the benefits of high-dose steroid treatment and outcome in biliary atresia (BA) remain controversial. In this study, we evaluated the impact of high-dose steroid therapy on the outcome of BA after the Kasai procedure. *Methods.* Intravenous prednisolone administration was started 1 week after surgery, followed by 8 to 12 weeks of oral prednisolone. Total bilirubin (TB) levels (3, 6, and 12 months after surgery), early onset of cholangitis, and two-year native liver survival were evaluated. *Results.* 53.4%, 56.9%, and 58.1% of the patients in the high-dose steroid group were jaundice-free 3, 6, and 12 months after surgery, respectively; these values were significantly higher than the 38.7%, 39.4%, and 43.3% of the low-dose steroid group. One year after surgery, the incidence of cholangitis in the high-dose group (32.0%) was lower than that in the low-dose group (48.0%). Infants with native liver in the high-dose group had a better two-year survival compared to those in the low-dose steroid group (53.7% versus 42.6%). *Conclusions.* The high-dose steroid protocol can reduce the incidence of cholangitis, increase the jaundice-free rate, and improve two-year survival with native liver after the Kasai operation.

## 1. Introduction

Biliary atresia (BA) is a progressive fibroobliterative disease of the biliary tract occurring in infants [1]. The survival rate in BA infant patients with native liver is less than 10% at 2 to 3 years following diagnosis without certain form of biliary drainage procedure [2]. In recent years, some studies have demonstrated improved outcome with routine postsurgery steroid therapy [3–7]. These studies used different maximum doses of steroids (1–10 mg/kg/d), a distinct dose-tapering schedule, and various treatment times (generally 1–3 months). Some of these studies showed that steroid therapy was not beneficial for BA patients [8, 9]. Due to the distinct regimens used and discrepancy of results obtained, the efficacy and safety of corticosteroids in improving biliary drainage remain controversial.

Since 2007, our institution had started to treat BA patients who received the Kasai operation with a high-dose steroid protocol. We also used antibiotics and ursodeoxycholic acid to prevent cholangitis and stimulate bile flow as well as bile drainage [10–12]. To evaluate the efficacy and safety of high-dose steroid treatment in BA patients, we retrospectively collected data from our hospital since 2007 and compared 2 different post-Kasai procedure regimens: (1) with low dose, short duration, and short antibiotic therapy versus (2) with high dose, long duration, and long antibiotic therapy. The current study aimed to review the current experience and lay the basis for future prospective randomized trials of high-dose steroid regimen in BA therapy.

## 2. Materials and Methods

### 2.1. Recruitment and Follow-Up

**Figure 1:**
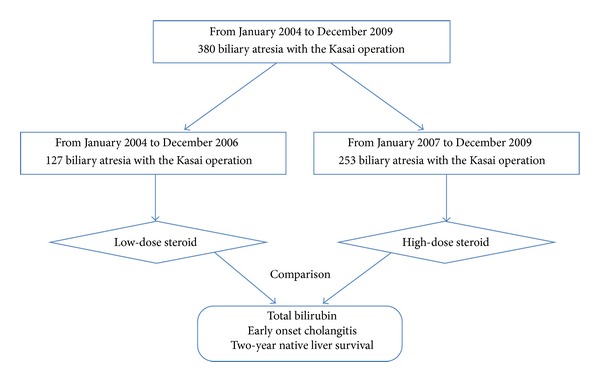
A flow chart of the patient groups and treatments.

A retrospective study of prospectively collected data was performed on all children (*n* = 380) who underwent the Kasai operation for treatment of BA from January 2004 to December 2009 in the Children's Hospital of Fudan University. All study protocols were approved by the Institutional Review Board at the Children's Hospital of Fudan University, IRB 2010167. The patient groups and treatments were designed as shown in Figure 1. Patients were followed for two years or until loss of native liver as a result of transplantation or death. The Kasai procedure was performed by a single physician using a well-established technique [13, 14]. The diagnosis of BA was confirmed by cholangiography and histological examination of resected biliary remnants. Cholangitis was diagnosed on the basis of the occurrence of fever, patient blood biochemistry data, and the decrease in bile secretion as detected by fecal color change. We reviewed the medical charts and analyzed for sex, age, associated abnormalities, operative age and usage of steroids, antibiotics, and ursodeoxycholic acid. Follow-up data were collected from parents, who were asked to provide answers to a detailed questionnaire either by telephone or in person in the clinic.

### 2.2. Treatment and Data Collection

Of the 380 treated patients, 127 children (from January 2004 to December 2006) received a low-dose steroid protocol and 253 children (from January 2007 to December 2009) were treated with a uniform postoperative protocol of high-dose steroids, long-term intravenous antibiotics, and ursodeoxycholic acid. In the groups receiving high-doses of steroids, intravenous prednisolone (taper of 4 mg/kg, 3 mg/kg, 2 mg/kg every three days) was started one week after surgery, followed by 8 to 12 weeks of oral prednisolone, which started at 4 mg/kg every other day and was gradually tapered until patients became jaundice-free (direct bilirubin <20 *μ*mol/L). This high-dose steroid protocol also included intravenous antibiotics (cefoperazone, 50–100 mg/kg/d divided every 8 hours plus metronidazole, 15–20 mg/kg/d twice a day) for 4 weeks, followed by 8 to 12 weeks of oral antibiotics (cefradine capsules, 25 mg/kg/d one time a day). This protocol also included oral ursodeoxycholic acid (20 mg/kg/d) for 12 to 24 months.

Children in the low-dose steroid group received 4 mg prednisolone/kg/d initially which was tapered to 2 mg/kg/d over one to two weeks. Intravenous antibiotics (cefoperazone, 50–100 mg/kg/d divided every 8 hours plus metronidazole, 15–20 mg/kg/d twice a day) were used for 1 week after surgery. Children in this group also received oral ursodeoxycholic acid (20 mg/kg/d) for 12 to 24 months.

### 2.3. Statistical Analysis

Statistical analysis was performed with SPSS 13.0 for Windows. The Pearson's chi-square test or Fisher's exact test was used to compare qualitative variables. Quantitative variables were analyzed by the Student's *t*-test. All data were reported as mean ± SD. A value of *P* < 0.05 was considered significant.

## 3. Results

### 3.1. Clinical Characteristics of Subjects

During the study period, 380 (163 males, 217 females) children underwent the Kasai procedure. Among them, no family history was found and 54 (14.2%) children had at least one abnormality (BA splenic malformation syndrome or other abnormalities). The average birth weight in the low-steroid (127) and high-steroid (253) groups was 3390 ± 438 g and 3420 ± 397 g, respectively. Twelve children in the low-dose steroid group and 7 in the high-dose group were lost to follow-up after 12 months and were included in the analysis of the postoperative bilirubin level and incidence of cholangitis but not in the analysis of the survival with native liver rates.

### 3.2. Age and Two-Year Survival with Native Liver

The average age at surgery in the low-steroid group (74 ± 31 days) was higher than that in the high-steroid group (66 ± 24 days, *P* = 0.032). Among the children (361) who underwent the two-year follow-up, the survival with native liver rate of children who received the Kasai operation at an age of <60 days was 55.1% (43/78). The survival with native liver rate dropped slightly in infants who received the Kasai operation between 60 and 90 days (48.9%, 71/145) and above 90 days (44.2%, 61/138). However, no significant difference was found among the different age groups (*P* > 0.05) (Table 1).

### 3.3. Outcome of Children Receiving Low-Dose and High-Dose Steroids

Three months after surgery, more children in the high-dose group (53.4%, 135/253) had normal bilirubin (serum total bilirubin ≤20 *μ*mol/L) compared with those in the low-steroid group (38.7%, 49/127; *P* = 0.007). At 6 and 12 months after surgery, the number of children with normal bilirubin also showed significant differences between the two groups (56.9%, 144/253 versus 39.4%, 50/127; *P* = 0.001) and (58.1% 147/253 versus 43.3%, 55/127; *P* = 0.001), respectively. According to the diagnosis standard, 12 months after surgery, 48.0% (61/127) of children in the low-dose steroid group suffered from cholangitis but only 32.0% (81/253) of those in the high-dose steroid group had cholangitis. The two-year survival with native liver rate in the high-dose steroid group was 53.3% (131/246). The survival with native liver rate dropped markedly in children who received low-dose steroids (38.3%, 44/115). There was a significant difference between the two groups (*P* = 0.001) (Table 2). However, no significant difference was found among the different time of Kasai operation groups (≤60 days, 61–90 days, and >90 days) (*P* > 0.05) (Table 3). Hemorrhage of the digestive tract happened in only one child in the high-dose steroid group after two weeks of intravenous steroid implementation and continued steroid therapy after bleeding arrest. No other major side effects (Cushing's syndrome, arterial hypertension, adrenal insufficiency, spontaneous fractures, abdominal wound infections, perforation of upper digestive tract, and anastomotic leakage) were found in our study.

## 4. Discussion

Begun as a desperate attempt to treat refractory ascending cholangitis in infants with BA following the Kasai operation, the use of steroids has evolved into a commonly used post-HPE therapy believed to improve clinical outcomes in BA. Karrer and Lilly [15] proposed using a very high-dose “blast-” type steroid treatment, which is supported by potential choleretic and anti-inflammatory properties that may benefit children with cholangitis. The choleretic effect of steroids involves the induction of the Na^+^-K^+^ ATPase, which increases canalicular electrolyte transport and stimulates bile flow independent of bile salt concentration [16]. In addition, when given at high doses, steroids have pronounced anti-inflammatory and immunosuppressive effects, decreasing edema and collagen deposition, inhibiting scarring, and arresting migration of infiltrating monocytes and lymphocytes [17].

The clinical use of steroids in BA has increased dramatically in recent years following the publication of a series of reports demonstrating better outcomes with routine post-HPE steroid therapy compared to a variety of historical or concurrent control groups [3–7]. Muraji and Higashimoto [6] described the use of steroids as an adjuvant medical treatment after Kasai portoenterostomy. An initial steroid dose of 4 mg/kg/d was tapered to 2 mg/kg/d over one to two weeks, and repeat “pulse” dose steroids were given if bile drainage tapered or cholangitis ensued. Ten of 14 patients (71%) became jaundice-free without liver transplantation compared with a 30% survival with native liver rate for the 10 patients treated before implementation of the adjuvant steroid regimen. Dillon et al. [3] were the first to advocate long-term, high-dose steroids and achieved equally impressive results, reporting 19 of 25 patients (76%) to be jaundice-free with their native liver at a mean follow-up of 4 years. Extending this work, the initial “pulse” dose of steroids (10 mg/kg/d) in Meyers's [5] report is higher than those used by Dillon, with a similar 2 mg/kg/d dose continued for several weeks. Ten of 14 patients (71%) in his study achieved appropriate and sustained bile flow.

In the current study, the low-dose steroid group received the same protocol as the patients studied by Muraji and Higashimoto [18]. However, in the high-dose group, the initial “pulse” dose of steroids is similar to that used by Dillon and lower than that used by Meyers. For a high-dose protocol, similar to that described in the study of Meyers and Dillon, oral prednisolone was continued for several weeks. In our current study, 3, 6, and 12 months after surgery, more children with the high-dose steroid therapy achieved appropriate and sustained bile flow compared with those in the low-dose steroid group. Similarly, the survival with native liver rate in the high-dose steroid group was much higher than that observed in the low-dose steroid group. The incidence of cholangitis in the high-dose steroid group was also much lower than that in the low-dose steroid group. The safety of high-dose steroid therapy in patient populations has been clearly shown in all four of these studies.

In our study, the two-year survival with native liver rate in the two groups (53.3% and 38.3%) is lower than that observed in the three studies mentioned above (71%, 76%, and 71%, resp.). However, those studies included lower numbers of children, making it difficult to compare survival with native liver rates between these previous reports and the current study. The survival with native liver rate in the high-dose steroid group in the current study (53.3%) is similar to that reported in a nationwide survey in Japan in 2004 (a group of 108 children received steroids at doses >4 mg/kg/d) [18].

One of the limitations of this study is retrospective collection of data, which is inferior to prospective randomized study. Although all surgeries were performed by a single physician with the same surgical technique and a larger number of patients were included in this study, whether the high-dose steroid protocol is primarily responsible for the improved outcome remains elusive, given that our current study has not been randomized or rigorously controlled for a host of potential confounding variables, and it is not empowered to definitively answer the question of causality. For instance, only the high-dose steroid group received long-term intravenous and oral antibiotics to lower the incidence of cholangitis. In the high-dose steroid group, the age at the time of surgery was slightly younger than that of the low-dose steroid group. Although some studies have concluded that children who received the Kasai procedure earlier always have a better prognosis, the age at time of surgery had no significant influence on survival with native liver in the present study [19–21].

Miyano and colleagues [22] showed that although about 70% of their patients had evidence of bile secretion immediately after surgery, only 32% maintained bile secretion after 5 years. However, children successfully treated with adjuvant steroid in Dillon's study [3] seem to maintain their bile flow for 5 years. The average follow-up of successfully treated patients in our current study is 2 years, and none of the children had significant growth retardation or portal hypertensive bleeding. Although we do not know whether or not the beneficial effects of adjuvant steroid therapy are caused by choleretic, immunosuppressive, or anti-inflammatory effects of steroids, the present study suggests that the high-dose steroid protocol could reduce the incidence of cholangitis, increase jaundice-free rate, and improve the two-year survival with native liver rate after the Kasai operation. Given the consistently promising results of the adjuvant steroid studies published recently, a randomized, controlled clinical trial is needed to further establish the utility of this protocol.

## Figures and Tables

**Table 1:** Age and two-year survival with native liver.

Age^1^ (days)	Number	Survival rate^2^
≤60	78	43 (55.1%)
61–90	145	71 (48.9%)
>90	138	61 (44.2%)

Total	361	175 (48.5%)

^1^At Kasai operation; ^2^number surviving >2 years with native liver.

**Table 2:** Outcome of children receiving low-dose steroid and high-dose steroid.

Outcome	Low-dose group	High-dose group	*P* value
Jaundice-free 3 months with native liver	38.7% (49/127)	53.4% (135/253)	<0.01
Jaundice-free 6 months with native liver	39.4% (50/127)	56.9% (144/253)	<0.01
Jaundice-free 12 months with native liver	43.3% (55/127)	58.1% (147/253)	<0.01
Incidence of cholangitis in 1 year	48.0% (61/127)	32.0% (81/253)	<0.01
Two-year survival with native liver rates	38.3% (44/115)	53.3% (131/246)	<0.01

Note: (1) Twelve children in the low-dose steroid group and 7 in the high-dose group were lost to follow-up after 12 months and were included in the analysis of the postoperative bilirubin level and incidence of cholangitis but not in the analysis of survival with native liver rates.

(2) Jaundice-free: direct bilirubin <20 *µ*mol/L.

**Table 3:** Outcome of children receiving high-dose steroid in different time of Kasai operation.

Outcome	≤60 days	61–90 days	>90 days	*P* value
Jaundice-free 3 months with native liver	25/48 (52.1%)	59/98 (60.0%)	51/107 (47.7%)	>0.05
Jaundice-free 6 months with native liver	29/48 (60.0%)	63/98 (64.3%)	52/107 (48.7%)	>0.05
Jaundice-free 12 months with native liver	31/48 (64.6%)	66/98 (67.3%)	50/107 (46.7%)	>0.05
Incidence of cholangitis in 1 year	14/48 (29.2%)	23/98 (23.3%)	44/107 (41.1%)	>0.05
Two-year survival with native liver rates	31/47 (65.9%)	51/95 (53.7%)	49/104 (47.1%)	>0.05

Note: (1) Seven children in the high-dose group were lost to follow-up after 12 months and were included in the analysis of the postoperative bilirubin level and incidence of cholangitis but not in the analysis of survival with native liver rates.

(2) Jaundice-free: direct bilirubin <20 *µ*mol/L.

(3) *P* value > 0.05: no significant difference was found among the different time of Kasai operation groups.
